# Clinical outcome of endoprosthetic replacement for failed treatment of intertrochanteric fractures: A retrospective case series

**DOI:** 10.12669/pjms.292.2964

**Published:** 2013-04

**Authors:** Wei Feng, Ting Hao, Wan-lin Liu, Yan-fei Jia, Zeng-tao Hao, Sheng-Bin Bai

**Affiliations:** 1 Wei Feng, Department of Orthopedics, The Second Affiliated Hospital, Inner Mongolia Medical University, Hohhot, China.; 2 Ting Hao, Department of Orthopedics, The Second Affiliated Hospital, Inner Mongolia Medical University, Hohhot, China.; 3 Wan-lin Liu, Department of Pediatric Orthopedics, The Second Affiliated Hospital, Inner Mongolia Medical University, Hohhot, China.; 4 Yan-fei Jia, Department of Orthopedics, The Second Affiliated Hospital, Inner Mongolia Medical University, Hohhot, China.; 5 Zeng-tao Hao, Department of Orthopedics, The Second Affiliated Hospital, Inner Mongolia Medical University, Hohhot, China.; 6 Sheng-Bin Bai, Department of Anatomy & Neurobiology, Xiangya School of Medicine, Central South University, 78 Xiangya Road, Changsha, China.

**Keywords:** Failure treatment of intertrochanteric fracture, Endoprosthetic replacement, Functional assessment

## Abstract

***Objective:*** The treatment methods for the failed internal fixation in elderly patients suffering from several osteoporostic fractures are still inconclusive. We aimed to evaluate the clinical effects of endoprosthetic replacement for failure treatment of intertrochanteric fracture.

***Methodology:*** A total of 13 patients with failed internal fixation for intertrochanteric fracture were collected between January 2002 and October 2009. All of them were treated with endoprosthetic replacement and followed up till October 2010. Four of them received total hip replacement and the remained nine received artificial bipolar femoral head replacement. Clinical and functional outcomes of patients were assessed.

***Results:*** Of 13 patients, nine were females and four were males with the mean age of 76.5 years (SD, 11.7, range, 58-92 years) at the time of fracture. The average time of operation and follow-up was 124 minutes (89-187minutes) and 31 months (14-68 months), respectively. The average blood loss during the operation was 631 ml (450-1560 ml). All patients showed good pain relief and functional improvement. Final post-operative Harris and WOMAC scores were significantly improved from pre-operative levels (*P*<0.05). Only five patients showed operative complications.

***Conclusions:*** Our finding indicated that endoprosthetic replacement is an effective salvage procedure for failed internal fixation of intertrochanteric fracture in elderly patients with effective pain relief and functional improvement as well as few serious complications.

## INTRODUCTION

Hip fractures are the leading cause of death and disability among the elderly, of which about 50% are intertrochanteric fractures. A large percentage of intertrochanteric fractures are unstable and occurred in women.^1^^,^^2^ However, most elderly persons with intertrochanteric fractures are accompanied with osteoporosis. The severe osteoporosis contributes to thrypsis, and thus reduces the successful of operation and stability of the internal fixation. Osteoporosis could lead to slow fracture healing and long recovery time,^3^ delay fracture union or nonunion, and induce loosening, prolapsing, cutting or breakage of the internal fixation after operation.^3^ Recently, the most common useful treatment for intertrochanteric fractures is internal fixation, however, failure rate of the internal fixation for intertrochanteric fracture is ranged from 4% to 17% even with the locking plate,^3^^,^^4^ depending on the osteoporosis and patient age. 

The treatment methods for the failed internal fixation among elderly patients suffering from several osteoporostic fractures are still inconclusive.^5^^-^^7^ These elderly patients often are unable to co-operate with partial weight-bearing. Therefore, the use of endoprosthetic replacement (EPR), though not commonly used for intertrochanteric fractures, has been advocated by some studies when confronted with this kind of population.^8^^-^^10^ Previous studies indicated prosthetic replacement was an effective method for treatment of failed fixation of intertrochanteric fracture.^11^^-^^18^ The purpose of this study was to evaluate the clinical effects of endoprosthetic replacement for failure treatment of intertrochanteric fracture.

## METHODOLOGY

***Patients: ***Between January 2002 and October 2009, 13 patients who received endoprosthetic replacement were included in our study, **who** had this after failure of the intertrochanteric fracture treatment. All patients had received a primary operation with internal fixation, and no pathological fractures were included. The clinical data of patients were recorded, including sex, age at diagnosis, previous surgery, Evans classification, and surgery type (Table-I). Among 13 patients, nine were females and four were males with the mean age of 76.5 years (SD, 11.7, range, 58-92 years) at the time of fracture. The interval time from the fracture to the initial operation was at the range of 6.3 to 13.1 months, and the average time was 9.52 months. Four patients received total hip replacement and the remained nine patients received artificial bipolar femoral head replacement.

Of 13 patients, all of them showed the failure of dynamic hip screw (DHS) internal fixation. Among the patients with DHS failure, eight patients presented with nonunion with penetration of the lag screw, and two showed loosened and broken screw, and three presented avascular necrosis of the femoral head. The complications of patients included inability of walk, hip joint pains and limb shortening as well as abnormity.

**Fig.1:**
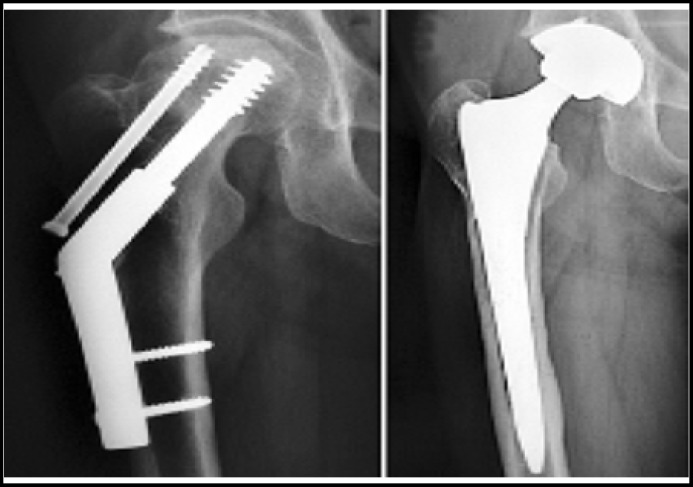
Postoperative X-ray of endoprosthetic replacement of patients with failed treatment of intertrochanteric hip fractures

***Surgical technique: ***General anesthesia was performed for all the 13 patients, and the adductor was selectively cut according to the hip joint motion. Operative incision was extended from the original operative scar to the lateral hip joint. Patients with unhealed trochanter and avulsion, the surrounding scar tissues and superfluous osteophyte were excised, and scar adhesion around joint was released from trochanter. Wire and tension band was used for fixation after trimming unhealed trochanter, and femoral head was taken out to trim for bone graft. Total hip joint replacement was performed for four patients, and artificial bipolar femoral head replacement was used for remaining eleven patients. After implantation, the joint stability was examined for all the patients. In the process of bone cement perfusion, the bone blocks of the fingers or femoral head were performed to block the original internal fixation screw-hole among patients with bone cement leakage. Post-operative X-ray of endoprosthetic replacement for patients with failed treatment of intertrochanteric hip fractures was showed in Fig.1.

***F*** ***unctional outcome assessment: ***The duration of surgery and pre**-**operative blood loss were recorded. The functional results were evaluated by using Western Ontario and McMaster Universities Osteoarthritis Index (WOMAC) scores and Harris Hip scores in all cases pre and post-operation.^19^^,^^20^ All the patients were followed up over three months after surgery and followed up till October 2010. 

Informed consent was obtained from all study subjects. The study was approved by the Ethics Committee of Inner Mongolia Medical University and Central South University.

***Statistical analysis: ***Statistical analysis was conducted by using SPSS 13.0. A *P* value of less than 0.05 was considered statistically significant. Comparison between Harris hip scores and WOMAC scores before and after surgery was conducted by paired T-test. 

## RESULTS

A total of two types of treatments were used in our study, including artificial bipolar femoral head replacement and hip replacement. The average time of follow-up among 13 patients was 31 months (14-68 months). No patient was lost to follow-up in our study. By Evans Classification, three patients were type II and the remained ten patients were type I for intertrochanteric fracture. The average operation time was 124 minutes (89-187minutes), and the average blood loss during the operation was 631 ml (450-1560 ml). Complications affect five patients, two patients suffered from postoperative dislocation, and the fractured trochanter of other two patients did not unite during reaming process, which could induce a weak abductor muscle. Another one presented femoral calcar split during operation.

During the average 31 months follow-up, eight patients felt no pain in hips, three felt mild pain, and the other two felt slight pain postoperation. No patient was limited to bed or chair, and only one patient needed an ambulatory aid for walk. The remained 12 patients did not need any assistive device for walk.

**Table-I:** Characteristics of patients with failed treatment of intertrochanteric hip fractures

*Patients Number*	*Age*	*Sex*	*Evans classification*	*Time from fracture to EPR (months)*	*Surgery type*
1	69	Female	I	11.8	Artificial bipolar femoral head replacement
2	74	Female	I	6.5	Total hip replacement
3	75	Female	I	10.9	Artificial bipolar femoral head replacement
4	78	Male	II	8.3	Artificial bipolar femoral head replacement
5	71	Female	I	9.5	Total hip replacement
6	73	Male	II	10.7	Artificial bipolar femoral head replacement
7	89	Female	I	10.2	Total hip replacement
8	75	Female	I	12.4	Artificial bipolar femoral head replacement
9	82	Male	I	15,7	Artificial bipolar femoral head replacement
10	78	Female	I	7.3	Artificial bipolar femoral head replacement
11	71	Female	I	12.2	Artificial bipolar femoral head replacement
12	92	Female	II	11.7	Total hip replacement
13	68	Male	I	12.2	Artificial bipolar femoral head replacement

The pre-and post**-**operative Harris and WOMAC scores at different follow-up periods are shown in Table-II. The average scores of Harris for artificial bipolar femoral head replacement and total hip replacement were 37(2.6) and 34(2.8) pre-operatively, respectively, and they increased to 81(4.6) and 85(4.8) post-operatively, respectively. Post-operative Harris scores were significantly improved compared to pre-operative by the two treatment methods (P<0.05). At the end of follow-up, the post-operative Harris scores did not significant raise by the two treatment methods. Similarly, post-operative WOMAC scores were significant improved at the time of one year follow-up when compared with pre-operation by the two treatment methods (P<0.05). However, the post-operative WOMAC scores were not significant improved at the end of follow-up.

**Table-II:** Mean scores of Harris and WOMAC by different surgery types

*Treatment method*	*Harris* * scores*			*WOMAC score*		
	*Pre* *-* *operation * *(SD)*	*Postoperation for one year * *(SD)*	*End of follow-up * *(SD)*	*Pre* *-* *operation * *(SD)*	*Postoperation for one year * *(SD)*	*End of follow-up * *(SD)*
Artificial bipolar femoral head replacement (N=9)	37(2.6)	81(4.6)*	82(4.8)	83(2.7)	42(5.1)*	41(5.2)
Total hip replacement (N=4)	34(2.8)	85(4.8)*	85(4.7)	85(2.5)	47(5.0)*	42(5.1)
Total (N=13)	36 (2.6)	83(4.7)*	83.5(4.8)	84(2.6)	45(5.1)	41.5(5.2)

*P value <0.05 when comparing Harris or WOMAC scores between postoperation and peroperation

## DISCUSSION

Failure treatment of intertrochanteric hip fractures usually induce severe functional disability and pain, especially for severe osteoporosis older people.^1^^,^^2^ Our study indicated endoprosthetic replacement is an effective salvage procedure for failed internal fixation of intertrochanteric fracture in elderly patients, and this method would gain effective pain relief and functional improvement as well as few serious complications.

The average time of surgery was 124 minutes (89-187minutes), which was longer due to removal of internal fixation device during surgery. The blood loss during operation was 631 ml, which was much higher than internal fixation surgery due to the fact that internal fixation device must be exposed by dissecting the old scar.

The most common complications were dislocation and fractured greater trochanter. It is reported that 1-20% of patients would suffer from dislocation.^11^ Problem with dislocation is thought to be the result of the extensive soft tissue resection around the hip that creates great difficulty in repairing the capsule and abductor lever arm. This kind of complication showed in two cases in our study. The two patients were given conservative treatment after presenting with dislocation. Fractured greater trochanter could induce the combination of severe osteoporosis, the stress riser effect of the big hole over the proximal part of the lateral cortex, and thus weaken the bone when the compression hip screw was removed, and incomplete healing of the previous fracture line. The surgery of removing the lag screw would weaken the greater trochanter. Exaltacion et al. reported 15 patients with failed hip fracture fixation who underwent hip arthroplasty had a great risk of fractured greater trochanter and nonunion, and the average operative time and blood loss were greater than primary surgery, which are in line with the finding of our study.^12^ Hoelsbrekken et al. indicates reducing the complication of fractured greater trochanter could be made by the method of dislocating the hip after the removal of the side plate during the process of removing the lag screw, and then cuting the femoral neck so as to remove the femoral head side.^13^ This procedure could reduce the complication of fractured greater trochanter and decrease the time during surgery.

Previous studies indicated elderly patients who received revision total hip arthroplasty with proximal femoral replacement prosthesis had better clinical efficacy.^14^^-^^17^ A recent study in Mexico by Archibeck et al. with 102 patients who underwent total hip arthroplasties suggests total hip arthroplasties is clinically successful for patients with failed internal fixation of hip fracture, of which 11% have early surgical complications related to this procedures.^14^ Another study with 20 patients after failed surgery of proximal femoral fractures indicates total hip arthroplasty is an effective treatment for failed osteosynthesis of hip fractures, and all the patients had pain relief and functional improvement, however, this method could induce high complication with disclocations postoperation and superficial infection.^15^ Haidukewych et al. reported older patients who underwent hip arthroplasty for failed treatment of itnertrochanteric hip fracture presented 100% survival rate of 7-year and 87.5% at 10-years, and almost 70% of the patients had good pain relief, and functional improvement as well as without limited to walk.^16^ The findings of our study are in line with previous studies. In our study, Harris score rose to from 34 preoperatively to 85 postoperatively during 31 months follow-up period. Similarly, a previous study conducted in Indian population reported a similar result, in which the mean Harris Hip Score increased from 32 preoperatively to 79 postoperatively at one-year interval.^15^

The findings of our study indicate endoprosthetic replacement is an effective salvage procedure for failed internal fixation of intertrochanteric fracture in elderly patients with effective pain relief and functional improvement. However, some serious complications such as dislocation and fractured greater trochanter are associated with endoprosthetic replacement, and further large study is warranted to clarify the effectiveness and complications of this procedure.

## Authors Contributions

WF: Designed the protocol, performed study and wrote the manuscript.

TH: Designed the protocol, performed study and prepared the final manuscript.

WLL: Designed the protocol and critically reviewed the manuscript.

YFJ: Performed study.

ZTH: Performed study.

SBB: Performed study.
